# *Wolbachia* endosymbionts induce neutrophil extracellular trap formation in human onchocerciasis

**DOI:** 10.1038/srep35559

**Published:** 2016-10-18

**Authors:** Francesca Tamarozzi, Joseph D. Turner, Nicolas Pionnier, Angela Midgley, Ana F. Guimaraes, Kelly L. Johnston, Steven W. Edwards, Mark J. Taylor

**Affiliations:** 1 Department of Parasitology, Liverpool School of Tropical Medicine, Pembroke Place L3 5QA, Liverpool, UK; 2 Department of Women’s and Children’s Health, Alder Hey NHS Foundation Trust, Eaton Road L12 2AP, Liverpool, UK; 3 Institute of Integrative Biology, University of Liverpool, Crown Street L69 7ZB, Liverpool, UK

## Abstract

The endosymbiotic bacteria, *Wolbachia,* induce neutrophilic responses to the human helminth pathogen *Onchocerca volvulus.* The formation of Neutrophil Extracellular Traps (NETs), has been implicated in anti-microbial defence, but has not been identified in human helminth infection. Here, we demonstrate NETs formation in human onchocerciasis. Extracellular NETs and neutrophils were visualised around *O. volvulus* in nodules excised from untreated patients but not in nodules from patients treated with the anti-*Wolbachia* drug, doxycycline. Whole *Wolbachia* or microspheres coated with a synthetic *Wolbachia* lipopeptide (*Wo*LP) of the major nematode *Wolbachia* TLR2/6 ligand, peptidoglycan associated lipoprotein, induced NETosis in human neutrophils *in vitro*. TLR6 dependency of *Wolbachia* and *Wo*LP NETosis was demonstrated using purified neutrophils from TLR6 deficient mice. Thus, we demonstrate for the first time that NETosis occurs during natural human helminth infection and demonstrate a mechanism of NETosis induction via *Wolbachia* endobacteria and direct ligation of *Wolbachia* lipoprotein by neutrophil TLR2/6.

Onchocerciasis (river blindness) is a parasitic disease affecting 37 million people worldwide, causing debilitating skin and eye pathology and accounting for an estimated global loss of 1 million Disability-Adjusted-Life-Years^1^. It is caused by the migrating progeny (microfilariae-mf) of the filarial nematode *Onchocerca volvulus*, released by adult females residing in subcutaneous nodules (onchocercomata). The inflammatory response to dying mf in the skin and eyes, and the release of their bacterial endosymbiont *Wolbachia,* is the basis of *Onchocerca* dermatitis and ocular keratitis (river blindness) immunopathology^2^.

Neutrophils are the major component of the early inflammatory infiltrate around damaged mf in the cornea and skin^3^,^4^,^5^,^6^. Their recruitment and activation, with subsequent development of tissue pathology or systemic adverse reactions to microfilaricidal drug treatment, depend on the presence of *Wolbachia*^3^,^4^,^5^,^7^,^8^,^9^,^10^. The diacylated N-terminal polypeptide (*Wo*LP) of the *Wolbachia* surface peptidoglycan-associated lipoprotein (*w*PAL) has been identified as the main trigger of the neutrophil inflammatory response during ocular keratitis via the activation of Toll-like Receptor (TLR)-2/6 on immune and stromal cells^11^. In addition, recent data illustrates that the *Wo*LP TLR2/6 ligation motif is sufficient to directly induce a range of activation phenotypes in human neutrophils *in vitro*, demonstrating a direct functional role in neutrophil-mediated inflammatory responses^12^.

In onchocercomata, neutrophils are an abundant leukocytic infiltrate and are frequently found adjacent or attached to *Wolbachia*-containing adult worms^13^. Neutrophil numbers are dramatically decreased within nodules derived from doxycycline treated patients, where *Wolbachia* are effectively depleted from nematode tissues, or in animals parasitized by *Onchocerca spp.* naturally devoid of the symbiont^13^,^14^. Nevertheless, neutrophils do not appear to be detrimental to the survival of *O. volvulus* adults, although defensin and calgranulins have been found on their surface, suggestive of neutrophil degranulation^13^,^15^,^16^,^17^,^18^.

A novel effector mechanism defined first in neutrophils^19^ has been identified as the formation of “neutrophil extracellular traps” (NETs), in a process referred to as NETosis. NET formation consists of the extrusion of nuclear content, decorated with granular and cytoplasmic proteins^19^,^20^,^21^. Extracellular traps (ETs) have latterly been observed from other granulocytes, such as eosinophils as well as mast-cells, and macrophages^22^. Although the range of biological functions of NETs continues to grow, a consensus has emerged that in infectious disease, NETs serve to limit microbial spread, by the entrapment of live microorganisms^22^. Moreover, it has been suggested that NETs may also limit collateral tissue damage in the context of inflammation by entrapping and degrading soluble pro-inflammatory cytokines and chemokines^23^.

In parasitological infectious disease, NET formation can be induced by a range of protozoan pathogens such as *Leishmania*, *Plasmodium, Toxoplasma*, *Eimeria, Besnoitia* and *Trypanosoma*^24^,^25^. The induction of NETs by multi-cellular, macroparasites has been reported *in vitro* and *in vivo* in experimental murine infection by larvae of the human gut nematode, *Strongyloides stercoralis*^26^,^27^, and eggs of the human blood fluke, *Schistosoma japonicum*^28^ where NETs are observed within the core of hepatic egg granulomas^29^. In filariasis, NETs release *in vivo* and *in vitro* has also been recently reported in the *Litomosoides sigmodontis* murine model with NETs observed in mouse skin at 6 hours post-infection^30^.

Because of the well-defined relationship between neutrophil recruitment and the *Onchocerca* endobacteria, *Wolbachia,* we hypothesised that NET formation was a possible phenotypic outcome of neutrophil recruitment in human onchocerciasis. Availability of nodule tissues from a placebo-controlled clinical trial^31^ where *Wolbachia* had effectively been depleted, allowed us to examine both evidence of NET production and dependency on *Wolbachia.* From our histological analyses we conclude that NETs are produced adjacent to the nematode in human onchocerciasis and their presence is strongly associated with neutrophils and *Wolbachia.* Our *in vitro* experiments demonstrate that whole *Wolbachia* or latex microspheres decorated with the *Wo*LP TLR2/6 ligand are sufficient to induce NETosis in human and mouse neutrophils, dependent on the TLR6 receptor.

## Results

### NETs release occur within human onchocercomata coincident with *Wolbachia* and neutrophil infiltrates

**Figure 1:**
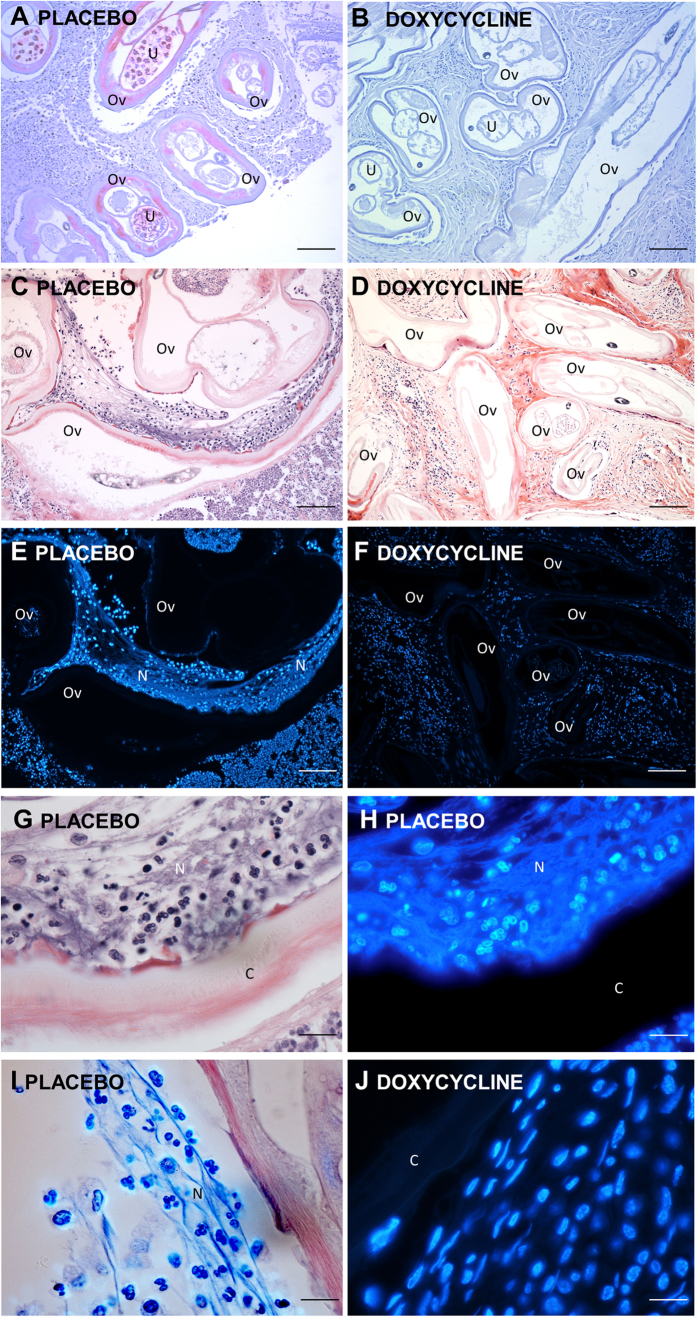
Representative histological sections of NET-like structures and neutrophil recruitment in placebo or doxycycline treated onchocercomata. Low-power (10x) magnifications of anti-*w*PAL staining (red) of *Wolbachia* endobacteria within adult nematode tissues from placebo treated individuals, (**A**) or in nodules from individuals treated with doxycycline (**B**). Low-power (10x) magnifications of H&E staining of placebo-treated (**C**) or doxycycline-treated (**D**) onchocercoma sections and sequential Hoechst staining (E&F). High-power (100x) magnifications of Hoechst (**G**) and H&E (**H**) stained section adjacent to adult worm cuticle. High-power (100x) magnifications of sections adjacent to adult cuticle, Hoechst/H&E sequentially stained derived from placebo (**I**) and Hoechst stained derived from doxycycline (**J**) treated onchocercomata. Key: (**C**), cuticle; N, NET-like structure; Ov, *Onchocerca volvulus,* U, uterus. (**A–F**) scale bar 500 μm; (**G–J**) scale bar 50 μm.

**Figure 2:**
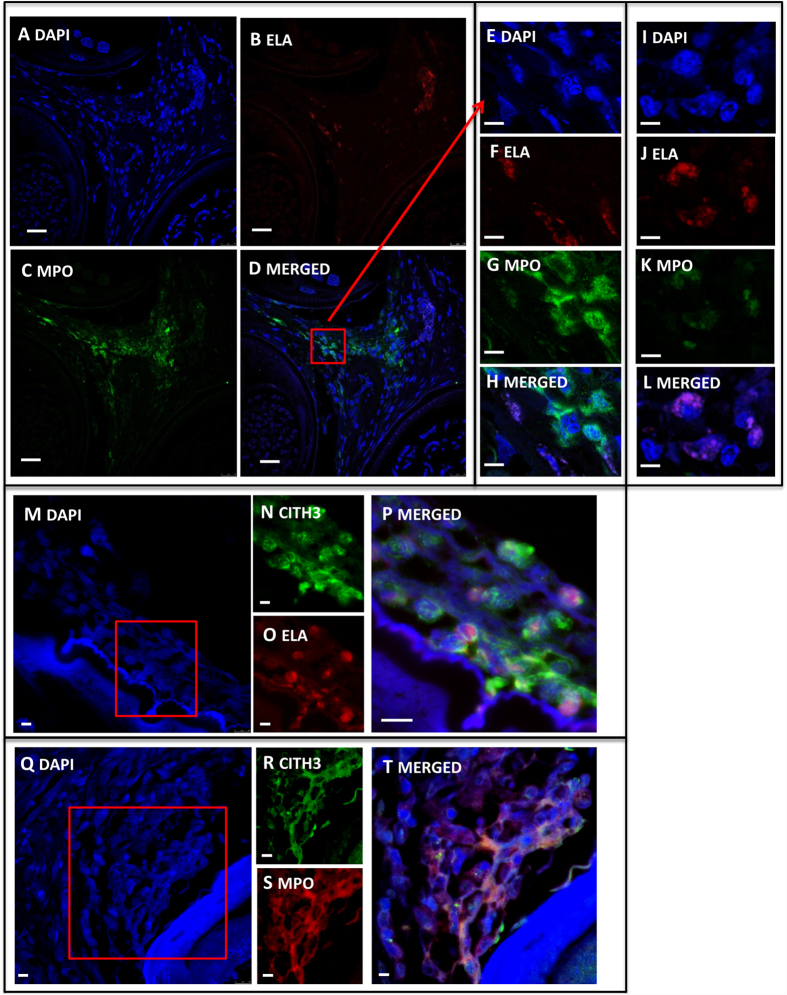
Representative immunofluorenscent histological sections of NETs in placebo treated onchocercomata. Low-power (50x) magnifications of DAPI (blue; **A**), neutrophil elastase (ELA, red; **B**), myeloperoxidase (MPO, green; **C**), and merged image (**D**) of a placebo-treated onchocercomata section. High-power (250x) magnifications of DAPI (blue; **E**), neutrophil elastase (red; **F**), myeloperoxydase (green; **G**) and merged image (**H**) of a specific area of the placebo-treated onchocercomata section from panels A to D (red frame) and showing extracellular release of elatase and myeloperoxidase from activated neutrophils. Conversely, resting neutrophils only displayed intracellular elastase and myeloperoxidase signals (DAPI in blue, elastase in red, myeloperoxidase in green and a merged image in panels **I**–**L** respectively). Presence of NETs was confirmed by colocalization of citrullinated histones, DAPI and either elastase or myeloperoxidase signals (panels **M** to **T**). High-power (150–250x) magnifications of DAPI (blue, **M**), citrullinated histones (CITH3, green; **N**), elastase (ELA, red; **O**) and merged image (**O**) of a placebo-treated onchocercomata. Following the same pattern, high-power (150–250x) magnifications of DAPI (blue, **Q**), citrullinated histones (CITH3, green; **R**), myeloperoxidase (MPO, red; **O**) and merged image (**T**) of another placebo-treated onchocercomata. (**A–D**) scale bar 100 μm. (**E–L**) scale bar 20 μm. (**M–T**) scale bar 50 μm.

**Figure 3:**
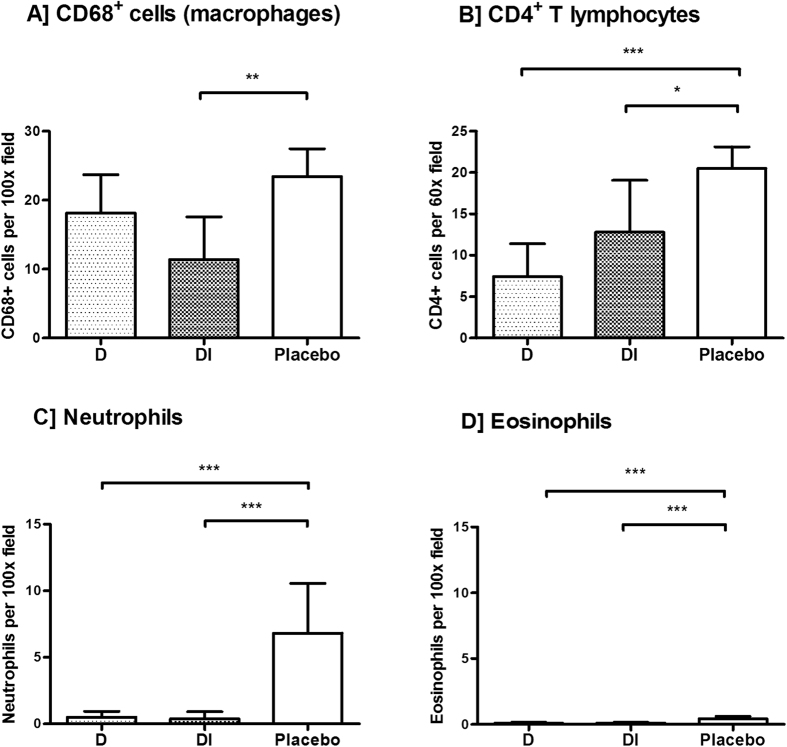
Cell populations in *O. volvulus* nodules. Nodules were from patients treated with doxycycline (**D**, *n* = 3 nodules), doxycycline + IVM (DI, *n* = 5 nodules), and placebo (*n* = 5 nodules). Cells were counted in 20 randomly selected fields of the indicated magnification within the whole nodule section. The distribution of the mean number of cells per field was positively skewed, therefore a Poisson regression model was applied and the corresponding p-value adjusted for clustering of replicates within nodules. Bar graphs represent number of cells (mean ± SD) per field. *p = 0.022; **p = 0.003; ***p ≤ 0.001.

All investigated onchocercomata from placebo (P) matching doxycycline +/− ivermectin-treated patients contained worms positive for *Wolbachia,* as assessed using immunohistochemistry (IHC) with a purified anti-*w*PAL IgG^11^,^32^, whilst all nodules derived from doxycycline (D) or doxycycline + ivermectin (DI) treated patients were *Wolbachia*-negative (Fig. 1A,B). This corroborated previous data from the same sample group examining the effective depletion of *Wolbachia* following doxycycline treatment via *Wolbachia* surface protein (WSP) IHC and *wsp* DNA copy number^31^ but in addition, confirmed the absence of the major *Wolbachia* neutrophil activating molecule, *w*PAL, from antibiotic-treated onchocercomata. In all P, but not D/DI onchocercomata, haematoxylin and Hoechst- or DAPI-positive extra-cellular material with a NET-like structure could be observed in zones adjacent to nematode cuticle (Figs 1C–J and 2). Neutrophils were frequently observed in placebo-treated (*Wolbachia*+) onchocercomata, around and in close contact with adult worms. These neutrophils were often found immersed in the NET-like material (Fig. 1C,E,G–I). A lack of eosinophilic granules on standard Haematoxylin-Eosin (H&E) staining was observed in the extracellular space around these cells, suggesting it was highly unlikely that those NET-like structures were degranulated eosinophils or mast cells (Fig. 1C,G,I). Absence of co-staining of NET-like structures with eosinophil major basic protein (MBP) and tryptase (TRYPT) (Supplementary Fig. S1), confirmed this hypothesis. On the contrary, these NET-like structures co-stained with myeloperoxidase (MPO), neutrophil elastase (ELA) and citrullinated histones (CITH3) indicating the presence of neutrophils undergoing NETosis in the NET-like DAPI-positive areas neighbouring the nematode cuticle (Fig. 2). Neutrophil number was dramatically decreased in doxycycline treatment groups, with virtual disappearance of these cells (*p* < *0.001* P *vs* D and DI, Fig. 3 and Table 1). In sections derived from doxycycline-treated onchocercomata, mononucleated cells were virtually the only cells surrounding worm tissues, although these were present in lower numbers compared to nodules from placebo patients, and there was no evidence of Hoechst-positive extracellular material (Fig. 1H). In particular, macrophages, identified as CD68+ cells, were present both in the centre and in the periphery of nodules, (*p* = 0.003 P vs D) and CD4+ T cells were mostly found in the centre of the nodules around the worms but not in close contact with them (*p* < 0.001 P vs D; *p* = 0.022 P vs DI). Eosinophils, although significantly reduced in D and DI compared to P nodules, were scant in all samples (Fig. 3 and Table 1).

### Whole *Wolbachia* induce NETosis in human neutrophils *in vitro*

**Figure 4:**
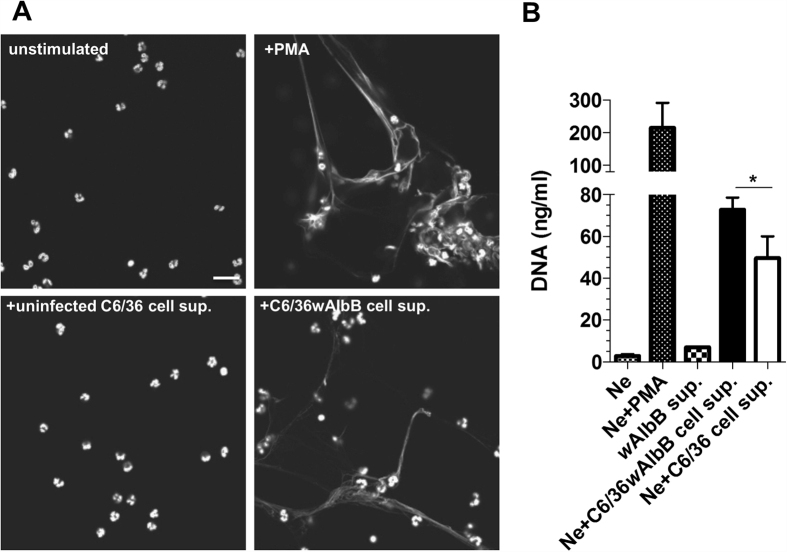
Induction of NETosis in human neutrophils incubated with whole *Wolbachia.* Representative micrographs of resting human neutrophil cultures or stimulated for 2 h with PMA, cell supernatant containing *Wolbachia* at an estimated ratio of 25 bacteria per neutrophil or matching concentration of *Wolbachia* free supernatant, subsequently stained with DAPI (white) **(A**) Scale bar 100 μm. Quantification of extracellular DNA stripped from cultures following stimulations with *Wolbachia* containing or *Wolbachia* free supernatant (**B**). Quantities of extracellular DNA released following *Wolbachia-*free and *Wolbachia-*containing cell supernatant stimulation of human neutrophils. Bars represent means (+SEM) of 6–9 replicates (2 for unstimulated Ne and Ne + PMA controls) pooled from 2 individual experiments. Matching C6/36wAlbB cell sup volumes were measured for DNA content by picoGreen DNA assay in duplicate. Difference between quantities of extracellular DNA released following *Wolbachia-*free and *Wolbachia-*containing cell supernatant stimulation of human neutrophils was evaluated by Mann-Whitney test. Significance indicated *p < 0.05.

As the presence of neutrophils and NETs were associated with the presence of *Wolbachia* in onchocercomata, we investigated whether whole *Wolbachia* bacteria freshly isolated from the culture supernatant of a *Wolbachia* infected *Aedes albopictus* cell line (C6/36 [*w*AlbB]) induced NETosis when co-cultured with human neutrophils. Detection and quantification of *w*AlbB within supernatants was verified using qPCR of the single copy *Wolbachia 16S rRNA* gene. We found that neutrophils incubated for 2 h with cell supernatant containing *Wolbachia* bacteria at a 1:25 ratio optimally induced NETs, which were typically observed in 10–15% of the total culture area. NETosis was reliant on the presence of *Wolbachia* as parental uninfected C6/36 cell line supernatant (*w*AlbB-) added to neutrophil cultures failed to induce NETs. Corroborating the visualisation of NETs post *w*AlbB co-incubation, increased extracellular DNA was quantified when stripped from neutrophil cultures (*p* = *0.036 cf w*AlbB- exposed cultures, Fig. 4).

### *Wolbachia* lipopeptide coated micro-particles are sufficient to induce NETosis in human neutrophils *in vitro*

**Figure 5:**
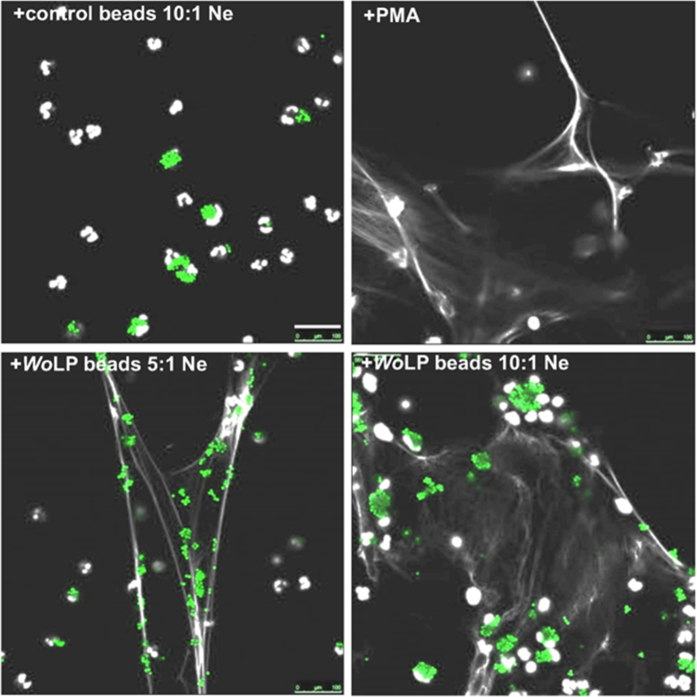
Induction of NETosis in human neutrophils incubated with *Wolbachia* lipopeptide (*Wo*LP)-coated microspheres. Representative micrographs of resting human neutrophil cultures or cultures stimulated for 2 h with PMA, *Wo*LP+ or *Wo*LP- microspheres at estimated ratios of 5 or 10:1 neutrophil (green), subsequently stained with DAPI (white). Images are representative of 2 individual experiments. Scale bar 100 μm.

The diacylated N-terminus of *Wolbachia* lipoprotein, *Wo*LP, has been defined as the predominant *Wolbachia* Pathogen-Associated Molecular Pattern, interacting with the host immune system via the TLR2/6-MyD88 signalling pathway^11^. Two native *Wolbachia* surface-associated lipoproteins have been characterised in both filarial and insect *Wolbachia*^11^,^32^. Filarial *w*PAL is abundantly expressed in female *O. volvulus* tissues (Fig. 1A and^11^) and has also been identified as a secreted *Wolbachia* protein in the secretome of the related human lymphatic filaria, *Brugia malayi*^33^,^34^. The *Wo*LP motif of *w*PAL is sufficient to activate multiple functions of neutrophils *in vitro*^12^. Therefore, we investigated whether *Wo*LP was a candidate *Wolbachia* molecule that interacted with human neutrophils to induce NETosis. Soluble *Wo*LP did not activate NETs in human neutrophils at all doses tested (0.001–5 μg/ml, data not shown). Because whole *Wolbachia* could induce NET formation, we hypothesised that particulates were necessary for NETosis, putatively via phagocytosis. To test this, 5 μm, fluorescently-labelled latex beads were coated with *Wo*LP and *Wo*LP+ or uncoated *Wo*LP- beads were used as a stimulus in human neutrophil assays. NETosis could be observed in approximately 10% of the culture area when isolated human neutrophils were exposed to *Wo*LP+ beads incubated for 2 h at ratios of between 10:1 and 5:1 beads:neutrophil (Fig. 5). No NETs were induced in neutrophil cultures incubated with *Wo*LP- control beads up to a ratio of 10:1 beads:neutrophil (Fig. 5).

### *Wolbachia* mediated NETosis is TLR6 dependent

**Figure 6:**
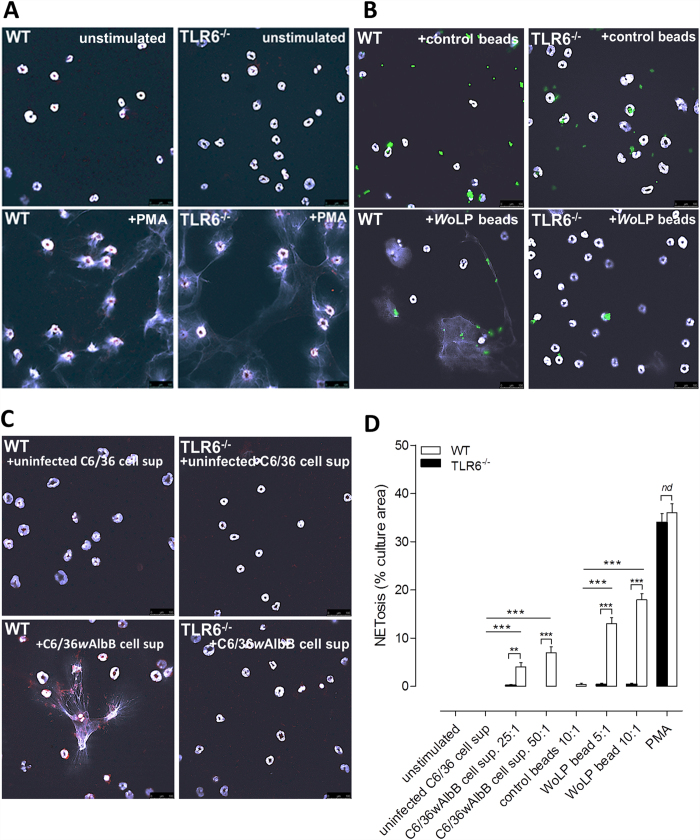
Induction of *Wolbachia* mediated NETosis is TLR6 dependent. Representative micrographs of resting murine wild type (WT) or TLR6 deficient (TLR6^−/−^) neutrophil cultures or cultures stimulated for 2 h with: PMA subsequently stained with DAPI (white) and neutrophil elastase (red) (**A**), *Wo*LP+ or *Wo*LP- microspheres (green), subsequently stained with DAPI (white) (**B**) cell supernatant containing *Wolbachia* or matching concentration of *Wolbachia* negative supernatant subsequently stained with DAPI (white) and neutrophil elastase (red) (**C**). Semi-quantitative scoring of percentage area of cultures containing DAPI+ NETs (**D**). Plots are mean % area NET +/−SEM from five cultures. Significance is indicated between negative controls and *Wolbachia* stimuli for WT cultures and between corresponding TLR6^−/−^ cultures (1way ANOVA ***P < 0.001, **P < 0.01, *P < 0.05). All data is representative of single neutrophil cultures derived from individual mice (n = 5 mice/group). Scale bar 100 μm.

Because filarial *Wolbachia* and *Wo*LP innate inflammatory activity, including neutrophil recruitment *in vivo,* has been shown to be TLR2/6 dependent^11^,^35^,^36^, we tested whether NETosis induction by *Wolbachia* also required an intact TLR2/6 receptor. For this we utilised TLR6 knock out mice that are non-responsive to cognate bacterial diacyl-lipoprotein ligands, including *Wo*LP^11^. Purified, isolated bone marrow mouse neutrophils from wild type (WT) C57Bl/6 inbred mice or C57BL/6 mice deficient in the TLR6 gene (TLR6^−/−^) were cultured with PMA, *Wolbachia* stimuli or controls for 2 h. NETs were visualised by DAPI staining of extracellular DNA and, where indicated, co-localised with anti-neutrophil elastase staining (Fig. 6). PMA induced vigorous NET production in both WT and TLR6^−/−^ neutrophils (mean culture area covered by NETs = 36%, WT *vs* 34%, TLR6^−/−^; Fig. 6A,D). Confirming *Wo*LP-specific NETosis in human neutrophils, we observed significant NET production in murine WT neutrophils (mean culture area covered by NETs = 18%, ratio of 10 *Wo*LP beads per neutrophil, *p* < 0.001 *vs* control bead cultures; Fig. 6B,D). We verified that the *Wo*LP-mediated NETosis visualised in human neutrophils was via the TLR2/6 receptor by determining that NET production was almost completely ablated in TLR6^−/−^ neutrophil cultures (mean culture area covered by NETs = 0.4%, *p* < 0.001 *vs* corresponding WT cultures; Fig. 6B,D). Corroborating data produced in human neutrophil cultures, whilst NETs were not observed when cultured with *Wolbachia-* supernatant, *Wolbachia*+ supernatant mediated significant NETs production in WT neutrophils (mean culture area covered by NETs = 7%, 50:1 *Wolbachia*:neutrophil ratio, *p* < 0.001 *vs* control; Fig. 6C,D). However, TLR6 deficiency made murine neutrophils unresponsive to *Wolbachia*+ supernatant in terms of NET production (*p* < 0.001 WT *vs* TLR6, 50:1 *Wolbachia*: neutrophil ratio).

## Discussion

We show, for the first time, that neutrophil infiltrates surrounding *O. volvulus* adult parasites in nodules are frequently contained within a DNA net-like material decorated with neutrophil-derived granule molecules and citrullinated histones as markers of NETosis. These structures were completely absent in *Wolbachia*-depleted nodules where the neutrophil infiltrate was replaced by NET-free mononuclear cells. These results are suggestive that neutrophil derived NETosis is a consistent feature of adult *O. volvulus* nematode infection of humans. Although other cell types may be a potential source of ETs, eosinophils appear to be virtually absent from these nodules, and ETs did not contain eosinophil or mast cell specific markers. To further investigate the ability of *Wolbachia* to drive NETosis, we exposed purified, peripheral blood human and bone marrow murine neutrophils to whole *Wolbachia* bacteria, soluble *Wo*LP, and latex beads coated with a synthetic lipopeptide, *Wo*LP, of the major *Wolbachia* TLR2/TLR6 ligand, peptidoglycan associated lipoprotein (PAL). Both whole *Wolbachia* and *Wo*LP-coated beads induced NETosis in a TLR6-dependent manner, whereas soluble *Wo*LP failed to trigger NETosis. Our results are consistent with a TLR2/6, phagocytosis-mediated induction of NETosis by liberated *Wolbachia* endobacteria, similar to that reported in response to *Staphylococcus aureus* infection in mice and described as “rapid vital NETosis”^37^,^38^. *S. aureus* whole bacteria induce rapid NETosis *in vivo* in a timeframe of  < 30 min-2 h. *S. aureus* vital NETosis is dependent on both phagocytosis and TLR2 because phagocytosis is not sufficient to induce NETosis in the absence of TLR2, and triacylated lipopeptides do not mimic the effect of the whole bacteria. Interleukin (IL)-8 has also been reported to induce NETosis^39^. Our previous work^12^ showed that *Wo*LP is able to induce IL-8 from human neutrophils, and so a contribution from autocrine IL-8 stimulation to *Wolbachia*-induced NETosis may further amplify and sustain NETosis.

Neutrophils play a pivotal role in *Wolbachia*-mediated pathogenesis of onchocercal disease manifestations. The TLR2/6 ligand, synthetic N-terminal diacylated lipopeptide *Wo*LP, of *Wolbachia* lipoprotein *w*PAL which constitutes the major proinflammatory molecule expressed by the endosymbiont, induces corneal neutrophil recruitment and activation in a mouse model of keratitis^11^. *Wo*LP is sufficient to activate multiple functions of human neutrophils such as chemotaxis, cytokine secretion, modulation of expression of surface adhesion molecules, oxidative burst and survival^12^. However, neutrophils do not appear to be directly detrimental to living adult worms in onchocercomata^13^ and their role in this stage of filarial parasitism is still unresolved. It has been suggested that *Wolbachia*-dependent recruitment of neutrophils around adult parasites in onchocercomata may facilitate nutrient uptake for reproductive function or to block the possibly deleterious recruitment and activation of eosinophils proximal to the worm cuticle^13^,^16^,^40^.

Our results from human onchocercomata tissues corroborate the identification of filarial-induced NETosis in skin-stage infections^30^. Filarial NET formation may be a host protective response provoked by endobacteria liberated with uterine and other secretion/excretions, which may entrap and limit the dissemination of *Wolbachia*. Also, appropriate NETosis enveloping viable female worms may aid, at the site of adult infection, in prevention of inflammatory damage induced by *Wolbachia* released from mf. Because NETs have been shown to effectively trap nematode larvae *in vitro*^27^, NETosis triggered by *Wolbachia* may also comprise an anti-parasitic response to limit the density of the tens of thousands of uterine-released mf produced daily by each female worm. Any decreases in migratory mf densities would limit the potential for immunopathology in the skin and eye and may also impact on the transmission potential of the parasite to infect the intermediate black fly vector. It has recently been shown that skin resident neutrophils can elicit anti-filarial responses^30^ and that NETs modify inflammation by entrapping soluble inflammatory mediators^23^. Therefore, *Wolbachia*-induced NETs may also have direct anti-inflammatory properties in modifying skin inflammation during onchocerciasis, and their release after drug-induced mf death deserves investigation.

The presence of NETs may also serve as a ‘cloaking device’ to decrease the penetrance of more damaging immune cells toward the cuticle surface of adult *Onchocerca.* In support of this, in closely related bovine onchocerciasis, *Wolbachia* depletion by antibiotic treatment triggers a replacement of neutrophils with degranulating eosinophils adjacent to the worm surface^16^,^40^. The alteration in granulocyte recruitment following loss of the endosymbiont precedes the time point when adult filariae lose viability^16^. Further, it is possible that the formation of NETs, at vulnerable points along the worm body, such as the uterine pore, may be important in preventing access by immune cells and the cytotoxic granule products they release. In addition to serving as a physical barrier to cellular penetration of the parasite cuticle, NETs may possibly down-regulate an orchestrated protective immune attack through the degradation of cytokine and chemokine paracrine signals^23^. These hypotheses promote the phenomenon of filarial NETs production via *Wolbachia* symbionts in a role consistent with a balanced, permissive parasitism whereby adult worms survive to reproduce yet immunopathology is limited.

## Methods

### Immunohistochemistry of onchocercomata

*O. volvulus* infected patients from North West Province, Cameroon were enrolled into a double-blind placebo-controlled randomized trial of doxycycline (6 weeks ± ivermectin 4 months after the start of treatment) and placebo^31^. Onchocercomata were surgically removed 21 months after treatment, fixed in 80% ethanol and embedded in paraffin. Sections of 4 μm mounted on Poly-L-lysine slides were rehydrated through serial dilutions of xylene and ethanol to water. After heat-induced antigen retrieval in 1 mM EDTA pH 8.0, presence of *Wolbachia* was assessed using rabbit polyclonal affinity-purified IgG against *w*BmPAL^11^ with Ultra-Vision ONE Detection System AP Polymer & Fast Red Chromogen (Thermo Scientific) following the manufacturer’s instructions. For CD68 and CD4 staining, after re-hydration and antigen retrieval, endogenous peroxidase was quenched by incubation in 3% H_2_O_2_ in methanol, before sections were blocked in TNB blocking buffer (Perkin Elmer). Primary antibodies were mouse anti-human CD4 IgG1 (clone 1 F6, Novocastra) and mouse anti-human CD68 IgG1 (clone KP1, Dako); secondary antibody was goat anti-mouse IgG-HRP conjugated secondary antibody (NEF822, Perkin Elmer). The Tyramide Signal Amplification (TSA) Plus FITC System (Perkin Elmer) was used as the revealing system. Slides were mounted in Pro-Long Gold anti-fade reagent or Vectashield Mounting Medium with DAPI. Slides from a nodule from placebo-treated patients were stained omitting the primary antibody as control for non-specific binding of the secondary antibody. A slide from a placebo-treated nodule stained omitting the primary antibody was included as a control. Hoechst 2 μg/ml (Invitrogen) was used to visualise DNA and cell nuclear shape. Sections were mounted with 1:1 glycerol:PBS and visualized using an Olympus BX60 microscope supporting Nikon DS-Fi1c camera with NIS Elements Imaging software (Nikon). To assess whether polymorphonucleated cells were neutrophils or eosinophils, standard Haematoxylin-Eosin (H&E) staining was carried out by serial passages in Harris Haematoxylin (Raymond A Lamb), 1% acid alcohol (1% v/v HCl, 70% v/v ethanol), Scott’s tap water (238 mM NaHCO_3_, 29 mM MgSO_4_), and 1% aqueous Eosin (Raymond A Lamb). To identify neutrophils as the polymorphonucleated cells in NET-like structures, after acquisition of images, the coverslip was carefully removed and on the same sections H&E staining was applied. Sections were mounted with Low Viscosity DPX mountant (Bios Europe) and images of the same areas were captured and merged with corresponding images of Hoechst-stained sections. To confirm the presence and the cellular origin of ETs in *Wolbachia*-containing onchocercomata, immunofluorescent staining was performed, using a specific marker of NETosis (i.e. citrullinated histone H3), specific markers of neutrophil ETs (neutrophil elastase and myeloperoxidase), and markers of mast cells (tryptase) and eosinophils (major basic protein) ETs. After re-hydratation and heat-induced antigen retrieval in target retrieval solution (Dako), onchocercomata sections were blocked in PBS-BSA 5%. Primary antibodies were polyclonal rabbit anti-neutrophil elastase (Abcam), polyclonal goat anti-myeloperoxidase (R&D), polyclonal rabbit anti-citrullinated histone H3 (Abcam), polyclonal rabbit anti-major basic protein (Abcam) and mouse anti-mast cell tryptase (clone AA1, Abcam). Secondary antibodies were goat anti-rat (AF594, Life Technology), goat anti-rabbit (AF488, Life Technology), donkey anti-goat IgG (AF594, Abcam) and goat anti-mouse IgG (Hparticular, macrophages, identified as L) Texas-Red (Thermo Fisher). Slides were mounted in Vectashield Mounting Medium with DAPI (Vector Labs). Non-specific binding control staining was performed by omitting primary antibodies in one slide per batch. Sections were visualized with a confocal laser-scanning microscope (Leica DM2500) and images captured with 40x or 63x objectives.

### Whole *Wolbachia* purification

*Aedes albopictus* mosquito cell lines C6/36 and C6/36 (*w*AlbB) were passaged in Leibovitz’s L-15 medium (Life Technologies) supplemented with 20% fetal bovine serum, 2% tryptose phosphate broth and 1% non-essential amino acids (Sigma Aldrich), as previously described^41^. Extracellular *Wolbachia* are routinely observed in culture supernatants of C6/36 (*w*AlbB) cells. To harvest *Wolbachia*-enriched supernatant, cultures were maintained without passage for 14–21 days. Supernatants were removed and centrifuged at 1000 g for 10 min to remove whole cells and debris. The resulting supernatant was stored on ice until use in the neutrophil culture experiments. Supernatant from the uninfected parental C6/36 cell line was collected using the same protocol and acted as a negative control. *Wolbachia* numbers present in each supernatant sample used in the neutrophil experiments were quantified using qPCR targeting the single copy *16S rRNA* gene^42^. DNA was extracted from 100 μl aliquots of supernatant using QIAamp DNA mini-kit reagents (Qiagen), eluting in a final volume of 50 μl and qPCR was performed as previously described^43^. Supernatant from the parental C6/36 line was confirmed to be *Wolbachia* free using this method.

### *Wo*LP microparticle preparation

Fluoresbrite 2 μm carboxylated latex microparticles (Polyscience) were covalently conjugated to *Wo*LP as per manufacturer’s instructions. Briefly, 12.5 mg beads were activated by water-soluble carbodiiamide (Polyscience), transferred into PolyLink coupling buffer (Polyscience) and incubated either with 500 μg *Wo*LP diluted in coupling buffer or matching volume of buffer (negative bead control). Beads were mixed for 1 h at room temperature before pelleting by centrifugation. Supernatant was retained to determine binding efficiency. Beads were transferred into storage buffer (Polyscience) and stored at 4 °C. Efficiency of *Wo*LP binding was determined by subtracting residual protein concentration in binding buffer from initial concentration, using colorimetric total protein estimation (BCA assay, Pierce). Efficiency of binding was typically >80%.

### Human peripheral blood neutrophil isolation

Peripheral blood was collected by venipuncture in lithium-heparin from healthy volunteers and neutrophils were isolated by Polymorphprep (Axis Shield) following manufacturer’s instructions. Contaminating red blood cells were lysed with 9:1 ammonium chloride lysis buffer (13.4 mM KHCO_3_, 155 mM NH_4_Cl, 96.7 μM EDTA) in RPMI 1640 culture media (Gibco). Cell viability was assessed by 0.2% Trypan Blue staining (Sigma Aldrich) and was always ≥98%. The purity of isolated neutrophils was assessed by rapid Romanowsky stain (HD Supplies) of cytospins (Cytospins 3, Shandon) followed by differential count of ≥700 cells by optical microscopy. The purity of isolated neutrophils was ≥97%.

### Mouse bone marrow neutrophil isolation

Homozygous Toll-like Receptor (TLR)-6^−/−^ mice on the C57BL/6 inbred background (a gift from Professor Akira, Osaka University, Japan) were re-derived by embryonic transfer into specific pathogen free (SPF) recipient female C57BL/6. The homozygous colony was maintained by sibling mating in SPF barrier facility at designated animal facility (Liverpool University of Biological Services Unit) as licensed procedure under the UK Animals Scientific Procedures Act. C57BL/6 wild type (WT) mice were purchased from Charles River UK. All experiments were performed in accordance with relevant guidelines and regulations. Approval was obtained for all animal experiments from the ethical committees of the University of Liverpool and LSTM. Experiments were conducted according to Home Office (UK) requirements. Bone marrow cells were collected from both hind legs’ femurs of 8–9-week-old WT or TLR6^−/−^ mice and were resuspended in PBS. Red blood cells were lysed by osmotic shock using 0.2% and 1.6% NaCl solutions. Cells were then subjected to a discontinuous 72–64% Percoll^®^ density gradient centrifugation (450 g) in 15 mL Falcon tubes for 30 minutes at 4 °C. Mature neutrophils were collected at the 72–64% interface (purity >93%, assessed by flow cytometry and by optical microscopy), washed three times in cold PBS then resuspended in PBS at the working concentration of 10^6^ cells/mL.

### Neutrophil cultures

For the whole *Wolbachia* bacteria-induced NETosis assay, neutrophil cultures were performed in 24-wells cell culture plates (Costar) at a density of 4×10^5^ cells/ml at 37 °C with 5% CO_2_ in RMPI media with 2% FCS (Sigma Aldrich). Cells were allowed to adhere for 1 h to a sterile round glass coverslip inserted in each well and followed by incubation for 2 h in the presence stimuli. Phorbol myristate acetate (PMA, 600 nM, Sigma Aldrich) was used as the positive control and culture media alone as the negative control. *Wolbachia*-containing supernatants of infected mosquito cells were used at a range of 2:1 to 300:1 ratio bacteria:neutrophil, while soluble *Wo*LP (synthetic 20-mer of the N-terminal region of *wBm*PAL di-palmitoylated at the N-terminal cysteine residue^11^, EMC Microcollections) was tested at a concentration range of 0.001–5 μg/ml. Equivalent volumes of supernatant from uninfected mosquito cell culture or culture media containing equivalent volumes of the *Wo*LP vehicle, Dimethyl Sulphoxide (DMSO, Sigma Aldrich) were also included as negative controls. *Wo*LP-conjugated fluorescent beads or negative control beads were added to neutrophils cultures at a concentration range of 5:1 to 100:1 microparticles per cell.

### NET detection

After incubation with stimuli or controls, neutrophil-coated coverslips were fixed in 4% paraformaldehyde in PBS, washed in PBS, permeabilized in 0.05% Tween20 in TBS for 1 min, and washed with TBS. For mouse derived neutrophils which were not stimulated with control or WoLP-conjugated beads, cells were then stained for neutrophil elastase (1:200, Abcam), incubated 1 h at room temperature, washed three times 5 min with 1xTBS, and then incubated for 1 h with a secondary antibody (Donkey anti-rabbit AlexaFluor488 IgG (Hparticular, macrophages, identified as L), 1:400, Life technologies). For all human or mouse derived neutrophils, DNA was then stained with 1 μg/ml 2-(4-Amidinophenyl)-6-indolecarbamidine dihydrochloride (DAPI) (Sigma-Aldrich), for 3 min. Cells were washed with TBS and viewed with a confocal laser-scanning microscope (Leica DM2500). Images were captured with a 40x objective.

### DNA quantification

Neutrophil cultures were digested using micrococcal nuclease (Sigma Aldrich) to dismantle the NET scaffold. The culture was incubated for 10 min at 37 °C in the presence of 0.5 nuclease units and enzymatic digestion was terminated by using 5 mM EDTA. After centrifugation at 200 g for 8 min, 100 μL of the cell-free supernatant was transferred into a flat-bottom 96-well plate for the quantification of double stranded DNA using the Quant-iT Picogreen assay (Invitrogen, Carlsbad, CA). One hundred microliters of Picogreen reagent was added to the samples, which were then incubated at room temperature in the dark for 4 min. Extracellular DNA was measured with a spectrofluorometer at 480-nm excitation and 520-nm emission.

### Statistics

For the statistical analysis of cellular infiltration in onchocercomata, cells were counted in 20 randomly selected 100x (neutrophils, eosinophils and CD68+ cells) and 60x (CD4+ cells) magnification fields of view of 5 nodules from 5 doxycycline-treated patients, 3 nodules from 3 doxycycline + ivermectin treated patients and 5 nodules from 5 placebo treated patients. The distribution of the mean number of cells per field was positively skewed, therefore a Poisson regression model was applied and the corresponding p-value adjusted for clustering of replicates within nodules. Significant differences between quantities of extracellular DNA released following *Wolbachia-*free and *Wolbachia-*containing cell supernatant stimulation of human neutrophils were evaluated by Mann-Whitney tests. Significant differences in NETs occurrence in murine neutrophil cultures were evaluated by 1-way ANOVA with Tukey post-hoc tests. A p-value ≤ 0.05 was considered significant. Computations were carried out in SPSS Statistics 20.0 (IBM) and Prism 5 (GraphPad).

### Study approval

Human parasite material was obtained from patients enrolled in a double-blind placebo-controlled randomized clinical trial conducted in Cameroon. The experimental protocol for this study was designed in accordance with the general ethical principles outlined in the Declaration of Helsinki. The trial was approved by ethics committees of the Tropical Medicine Research Station, Kumba, Cameroon, and the Research Ethics Committee of The Liverpool School of Tropical Medicine, Liverpool, UK. Written informed consent was obtained from all participants, with the exception of those who were illiterate, where a literate witness signed on behalf of the participant and the participant added a thumbprint. The trial is registered with the current controlled trials registry, no: ISRCTN48118452. Ethical approval for the storage and experimentation on nodule tissues was obtained from NHS National Research Ethics Service (09/H1001/81, Northwest 4 REC) and for the use of blood neutrophils from adult healthy volunteers by the Research Ethics Committee of the University of Liverpool, UK.

## Additional Information

**How to cite this article**: Tamarozzi, F. *et al*. *Wolbachia* endosymbionts induce neutrophil extracellular trap formation in human onchocerciasis. *Sci. Rep.* **6**, 35559; doi: 10.1038/srep35559 (2016).

## Supplementary Material

Supplementary Information

## Figures and Tables

**Table 1:** Statistical analysis of cell populations in *O. volvulus* nodules.

Treatment Group	Mean	Standard Deviation		IRR (Incident Rate Ratio) (95% Confidence Interval)	*p-*value
		Between nodules	Within nodules		
CD68^+^ macrophages					
P	23.44	4.02	12.18	P vs D 1.29 (0.93–1.79)	0.125
D	18.13	5.59	10.10	P vs DI 0.48 (0.30–0.78)	0.003
DI	11.38	6.20	0.76	D vs DI 0.63 (0.37–1.07)	0.087
CD4^+^ T lymphocytes					
P	20.5	2.62	11.1	P vs D 0.36 (0.22–0.58)	<0.001
D	7.42	3.99	12.6	P vs DI 0.62 (0.42–0.93)	0.022
DI	12.8	6.28	12.4	D vs DI 0.58 (0.31–1.09)	0.091
Neutrophils					
P	6.82	3.74	9.81	P vs D 13.37 (4.96–36.05)	<0.001
D	0.51	0.44	1.49	P vs DI 17.95 (5.61–57.43)	<0.001
DI	0.38	0.54	1.61	D vs DI 1.34 (0.27–6.61)	0.717
Eosinophils					
P	0.42	0.19	0.68	P vs D 4.67 (2.13–10.21)	<0.001
D	0.09	0.08	0.34	P vs DI 3.82 (1.93–7.56)	<0.001
DI	0.11	0.04	0.31	D vs DI 0.82 (0.34–1.97)	0.655

CD68^+^ macrophages, CD4^+^ T cells, neutrophils and eosinophils in *O. volvulus* nodules derived from placebo matching doxycycline + ivermectin treated (P, *n* = 5 nodules), doxycycline-treated (D, *n* = 3 nodules) or doxycycline + ivermectin treated (DI, *n* = 5 nodules) analyzed by a Poisson regression model. Cells were counted in 20 randomly selected fields (100x magnification for CD68^+^, neutrophils and eosinophils, 60x magnification for CD4^+^ cells) within the whole nodule section. The findings are reported as mean counts per group, with within-nodule and between-nodule standard deviations. Differences between groups are reported as incidence rate ratios (IRR) with their 95% confidence intervals; these intervals and the corresponding p-values are adjusted for clustering of replicates within nodules.
