# Supplementary material for: Wolbachia endosymbionts induce neutrophil extracellular trap formation in human onchocerciasis
Source: Sci Rep. 2016 Oct 18;6:35559. doi: 10.1038/srep35559 (PMC5067710; doi:10.1038/srep35559)
Supplement: Supplementary Information [file srep35559-s1.pdf]

Wolbachia endosymbionts induce neutrophil extracellular trap formation in human onchocerciasis

Francesca Tamarozzi[1#], Joseph D. Turner[1#], Nicolas Pionnier[1#], Angela Midgley[2], Ana F. Guimaraes[1], Kelly L. Johnston[1], Steven W. Edwards[3], and Mark J. Taylor[1*]

Supplementary Information

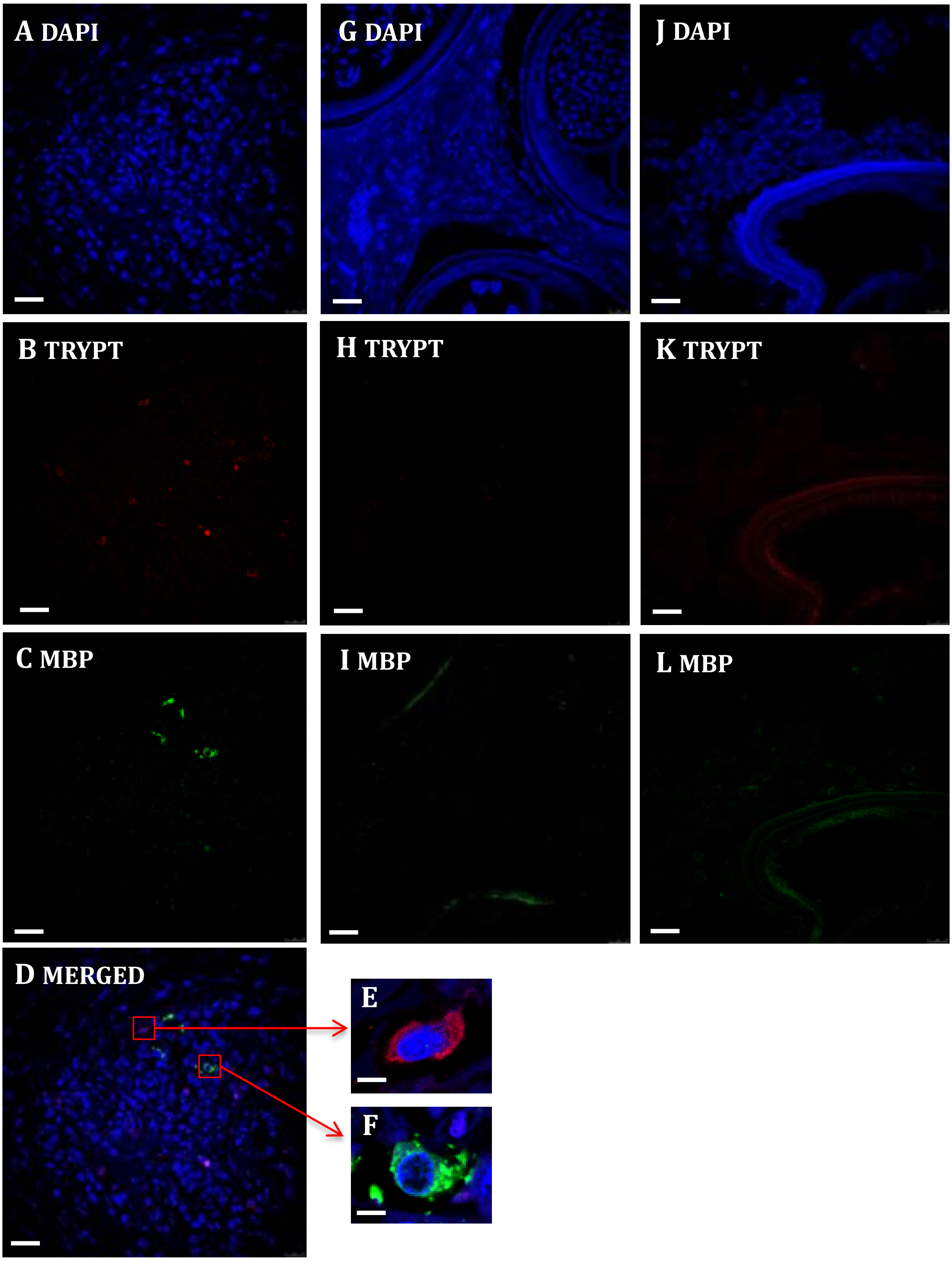

Supplementary Figure 1. Mast cells and eosinophils are not releasing ETs in placebo treated onchocercomata. Several mast cells and eosinophils were detected in placebo-treated onchocercomata sections but only in areas of large cellular

infiltrates (panels A to F) and not in ET-like areas in zones adjacent to nematode cuticle (panels G to L). Low-power (50x) magnifications of DAPI (blue; **A**), mast cell tryptase (TRYPT, red; **B**), eosinophil major basic protein (MBP, green; **C**), and merged image **(D)** of a placebo-treated onchocercomata section. Higher magnifications of red framed areas of the panel D are depicted in panels **E** and **F** showing specific staining for mast cells and eosinophils respectively. Images **G** to **I** (DAPI, TRYPT and MBP respectively) are captured from the same area of subsequent sections of the same onchocercomata of Figure 2 panels A to D. Images **J** to **L** (DAPI, TRYPT and MBP respectively) are captured from the same area of subsequent sections of the same onchocercomata of Figure 2 panels M to O. A-D scale bar 100 µm. E-F scale bar 20µm. G-L scale bar 100µm.
